# Epidermolysis Bullosa Simplex‐Severe Caused by *KRT5* p.Glu477Lys: Challenges Encountered in This High‐Risk Subtype

**DOI:** 10.1111/pde.70182

**Published:** 2026-09-04

**Authors:** Nataliia Zhovta, Joana Lanz, Bettina Hafner, Karin Willi, Sven‐Eric Baller, Stephanie Adzikah, Martin Theiler, Lisa Weibel, Isabelle Luchsinger, Kathrin Neuhaus, Simon Küpper, Christa Relly, Jörg Thomas, Eva Bergsträsser, Nicole Knöpfel, Agnes Schwieger‐Briel

**Affiliations:** ^1^ Dermatology Department, Pediatric Skin Center University Children's Hospital Zurich Zurich Switzerland; ^2^ Children's Research Center (CRC), University Children's Hospital Zurich University of Zurich Zurich Switzerland; ^3^ Clinic for Dermatology and Allergology, Kantonsspital St. Gallen St. Gallen Switzerland; ^4^ Division of Plastic and Reconstructive Surgery, Pediatric Skin Center University Children's Hospital Zurich Zurich Switzerland; ^5^ Neonatology Department University Children's Hospital Zurich Zurich Switzerland; ^6^ Division of Infectious Diseases and Hospital Epidemiology University Children's Hospital Zurich Zurich Switzerland; ^7^ Anesthesia Department University Children's Hospital Zurich Zurich Switzerland; ^8^ Pediatric Palliative Care Department University Children's Hospital Zurich Zurich Switzerland; ^9^ Department of Dermatology Medical Center University of Freiburg Freiburg Germany

## Abstract

Epidermolysis bullosa simplex‐severe (EBS‐severe) caused by *KRT5* p.Glu477Lys is a rare and particularly severe subtype associated with high neonatal morbidity and mortality. We report an infant who during the neonatal period required prolonged multidisciplinary intensive care for the management of several complications, including extensive wounds with blood loss leading to secondary anemia, failure to thrive with gastroesophageal reflux, respiratory distress with stridor and a necrotizing soft tissue infection, ultimately requiring surgical debridement and grafting. Pain control proved inadequate despite continuous morphine infusion, leading to initiation of methadone, which provided effective and stable analgesia. This case underscores the severe multisystem morbidity of *KRT5* p.Glu477Lys–associated GS‐EBS and underscores the role of methadone in effective pain management in critically affected EB neonates.

Epidermolysis bullosa simplex‐severe (EBS‐severe) caused by *KRT5* p.Glu477Lys is a rare but particularly severe subtype. This missense pathogenic variant affects the highly conserved KLLEGE motif of keratin 5 and has been implicated in a severe phenotype with high neonatal mortality [1, 2]. Herein, we describe a case of an infant with this genotype, who, during the neonatal period, required prolonged multidisciplinary intensive neonatal care for the management of several complications. These included extensive wounds with blood loss leading to secondary anemia, refractory pain despite opioid escalation, respiratory distress with stridor, failure to thrive with gastroesophageal reflux, and a deep soft tissue infection with septicemia requiring surgical wound management.

A full‐term female neonate, firstborn to healthy non‐consanguineous parents with an uneventful pregnancy, was delivered by cesarean section due to meconium‐stained amniotic fluid with a birth weight of 2500 g and Apgar 9/10/10. At birth, she presented with areas of absent skin of all distal extremities, large erosions and blisters over her trunk and severe involvement of the oral mucosa (Figures 1 and 2A). Genetic testing identified a *de novo* heterozygous missense variant in *KRT5* NM_000424.4:c.1429G>A, p.Glu477Lys.

**FIGURE 1:**
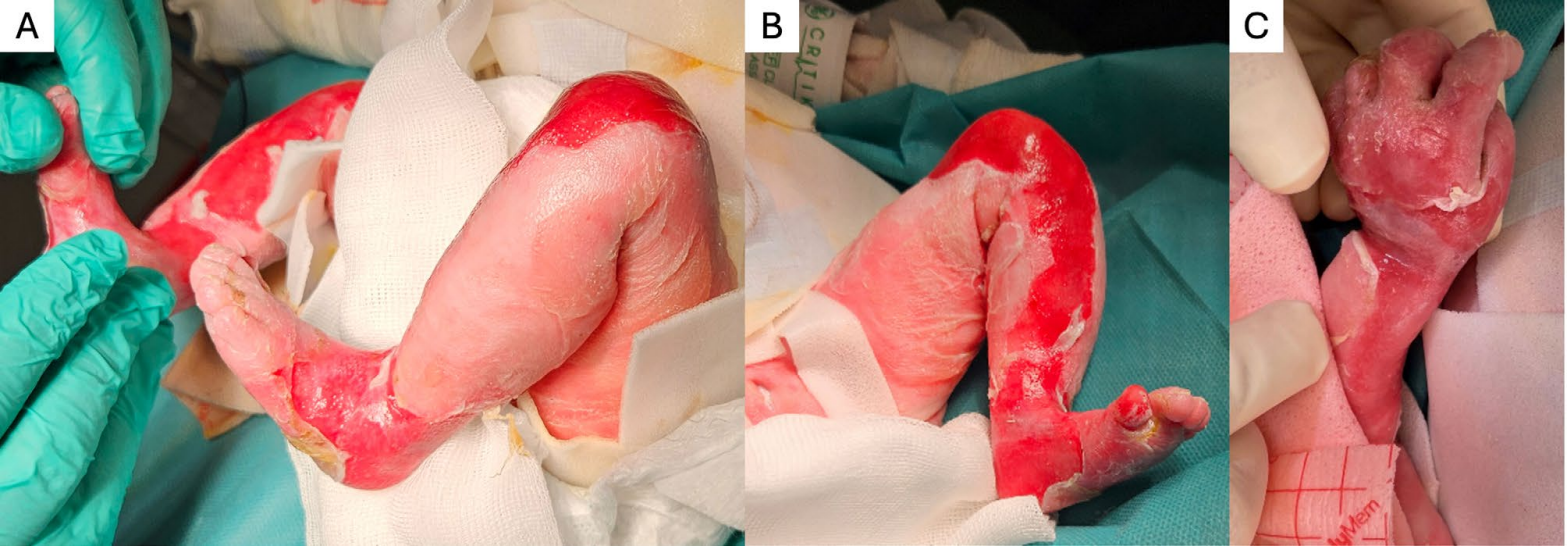
Clinical presentation showing large areas of absence of skin at birth involving all extremities (A–C).

**FIGURE 2:**
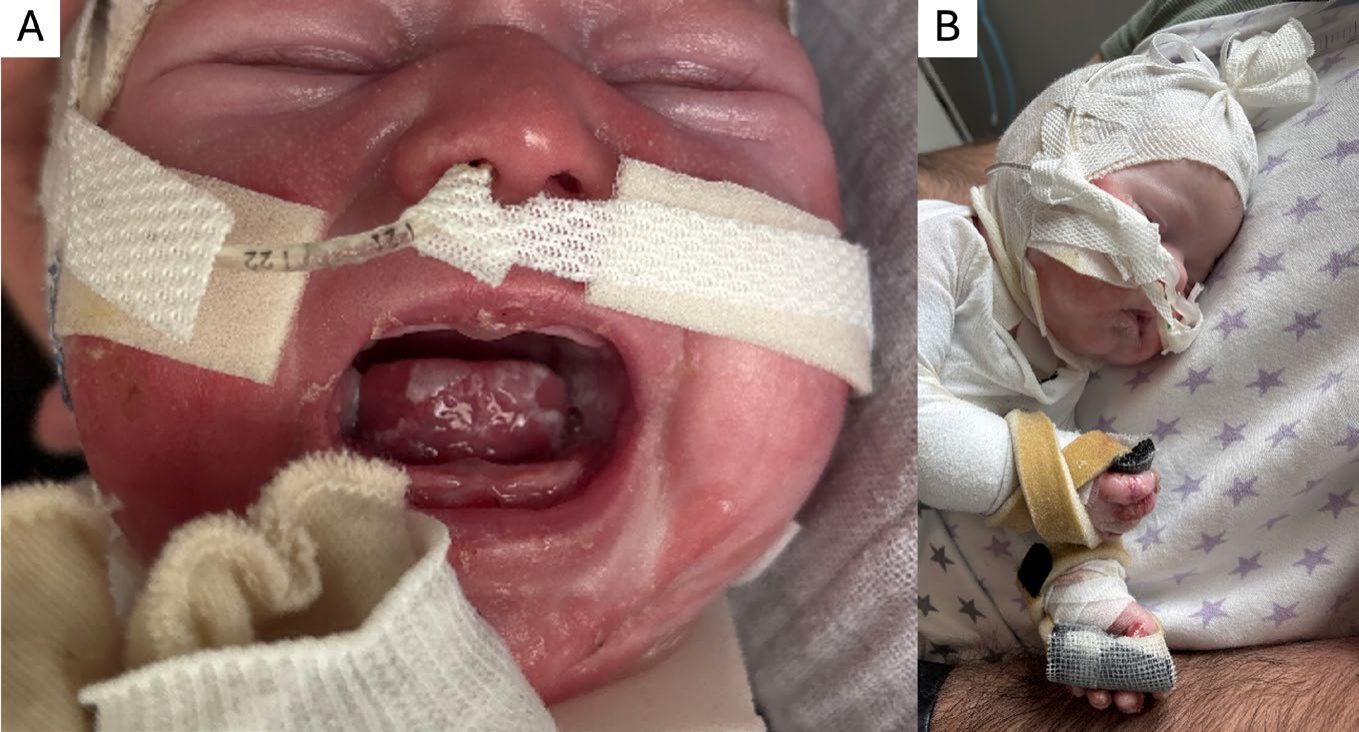
Severe involvement of the oral mucosa, with erosions on the ventral tongue and gingiva (A). Our patient with GS‐EBS under high‐flow oxygen therapy (B).

Initial boluses of morphine and ketamine used during dressing changes were ineffective. Subsequently, continuous morphine infusion (17 mcg/kg/h) and clonidine (1.8 μg/kg/dose every 4 h) were also ineffective. Therefore, at 5 weeks of age, methadone (0.05 mg/kg/dose every 8 h) was initiated while morphine was gradually tapered, rapidly providing effective and stable pain relief.

At birth, she had a hoarse cry and within weeks developed inspiratory and expiratory stridor along with increased airway secretions and lowered blood oxygen, requiring noninvasive respiratory support with high flow therapy (FiO_2_ 21% at 6 L/min; Figure 2B). Laryngoscopy was avoided due to the risk of mucosal injury. By 2 months of age, respiratory support was no longer required. Stridor subsided by 10 weeks of age but remained noticeable when the child was agitated until the age of 8 months.

Her clinical course was further complicated by fever and the development of large ulcerated areas of the skin on the left gluteal area (Figure 3A). A skin biopsy for tissue culture grew *Clostridium perfringens* , consistent with a deep necrotizing soft tissue infection, which required two rounds of surgical debridement with donor and synthetic (Novosorb BTM) skin grafting. A rectal tube was placed to minimize contamination. With thorough lubrication to avoid friction, it was well tolerated without apparent mucosal complications during the postoperative period (Figure 3B).

**FIGURE 3:**
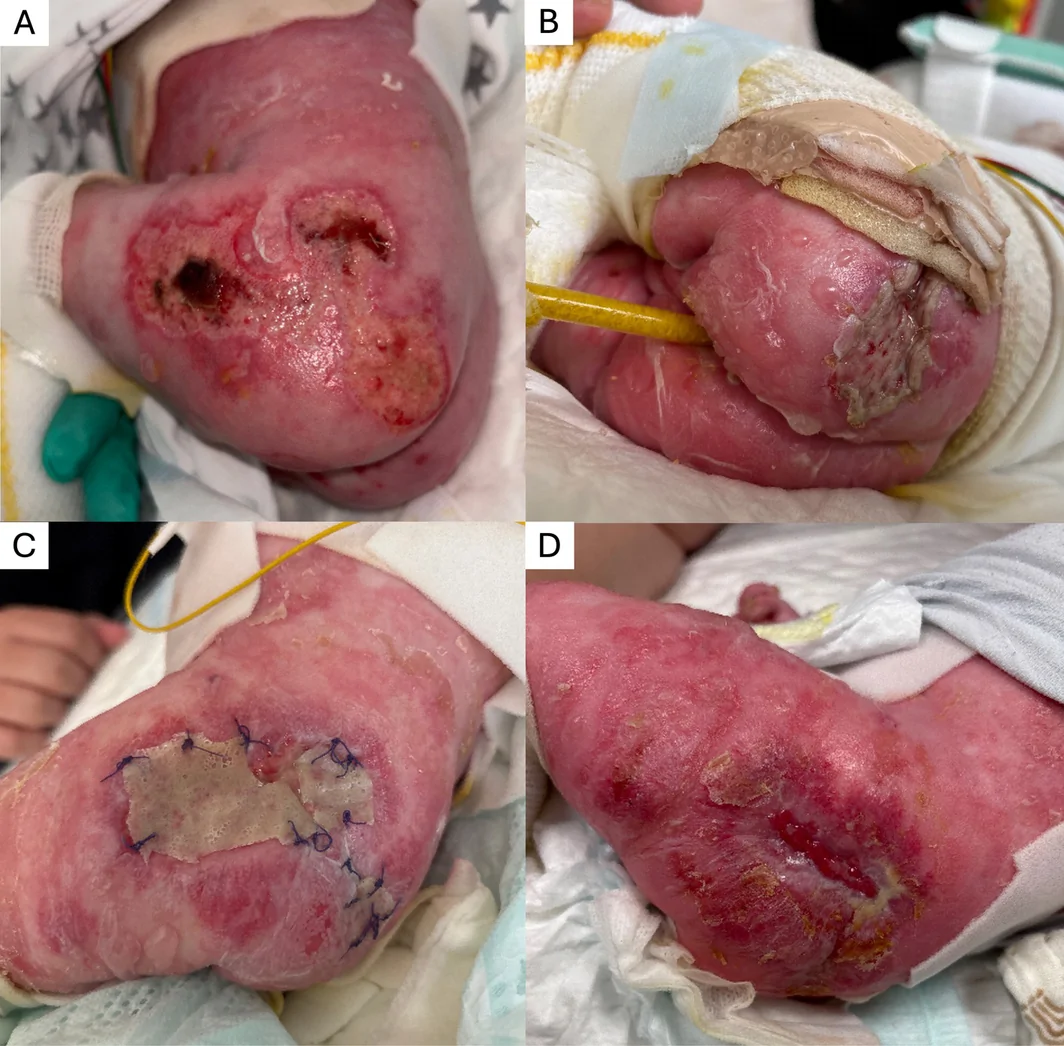
Large, ulcerated areas of the skin on the left gluteal area (A). After surgical debridement and temporary coverage using cadaver skin and rectal tube placement (B). After second debridement and wound coverage with Novosorb BTM (C). Clinical appearance after 3 weeks (D).

Over the course of her hospital stay, she required high caloric enteral nutrition initially through a nasogastric tube and later, due to gastric reflux and vomiting, a nasojejunal tube which allowed her to gain weight, without exacerbation of mucosal disease. Gastrostomy was recommended but declined by the family. She also developed acute anemia (lowest Hb level 66 g/L), secondary to blood loss during dressing changes, requiring two erythrocyte transfusions. At the most recent follow‐up at 8 months of age, the patient has shown an improvement in growth and weight gain and is currently between percentiles 3–10. She remains pain‐free with persistent skin fragility and has not developed new major complications.

Our case contributes to the growing body of evidence that EBS‐severe caused by *KRT5* p.Glu477Lys is associated with severe multisystem morbidity, characterized by early failure to thrive, respiratory failure, severe refractory pain, and in this case, necrotizing soft tissue infection. Previous reports have documented the poor prognosis associated with this genotype, with many affected neonates succumbing in the early postnatal period [1, 2].

Airway involvement with hoarse cry and stridor has been reported in severe junctional epidermolysis bullosa (JEB) and EBS with muscular dystrophy, and rarely in EBS‐severe [3]. In our patient, these symptoms ultimately led to respiratory exhaustion requiring prolonged high‐flow oxygen therapy for 4 weeks, after which respiratory function slowly improved and no further support was needed.

Nasogastric tubes are generally poorly tolerated in EBS‐severe due to mucosal fragility, with gastrostomy being preferred for longer‐term enteral feeding in these patients [4]. Rectal tube use is not well described in EBS‐severe; however, perianal blistering and constipation are recognized complications and invasive rectal interventions should be approached with caution [5]. In contrast, junctional epidermolysis bullosa (JEB) and dystrophic epidermolysis bullosa (DEB) are characterized by much greater mucosal and epithelial fragility, granulation tissue formation (JEB) and scarring (DEB); therefore, nasogastric tubes are discouraged in these forms [5]. This difference in tolerance to enteral tubes across EB subtypes is explained by protein expression in these anatomic areas [6].

This case also underscores that continuous morphine infusion may be insufficient to achieve adequate analgesia in neonates with severe EB subtypes. In our institution, pediatric palliative care is routinely involved in severely affected neonates, with a focus on symptom management, family support, and shared decision‐making, not being limited to end‐of‐life care. Pain management in neonates with EB is particularly challenging due to repeated dressing changes and mucosal involvement [7]. Morphine remains the most widely used opioid; however, it did not provide adequate pain relief in our patient, leading to the initiation of methadone. Methadone exhibits several pharmacological properties, including a long half‐life, combined μ‐opioid receptor agonist and N‐methyl‐D‐aspartate (NMDA) receptor antagonist activity, and reduced tendency to induce opioid tolerance, making it suitable for prolonged analgesia [8]. In EB, however, its use has been scarcely reported. We observed that in our case, methadone was a true game changer and provided effective and stable analgesia. We therefore propose its consideration as part of individualized pain management strategies for EB.

## Consent

The authors attest to obtaining written parent consent for all patient photographs or other identifiable material, with the understanding that this information may be publicly available.

## Conflicts of Interest

The authors declare no conflicts of interest.

## Data Availability

Data sharing not applicable to this article as no datasets were generated or analysed during the current study.
